# *Tetraedron minimum*, First Reported Member of Hydrodictyaceae to Accumulate Secondary Carotenoids

**DOI:** 10.3390/life11020107

**Published:** 2021-01-30

**Authors:** Philipp Doppler, Christoph Kornpointner, Heidi Halbwirth, Daniel Remias, Oliver Spadiut

**Affiliations:** 1 Research Division Biochemical Engineering, Institute of Chemical, Environmental and Bioscience Engineering, TU Wien, Gumpendorfer Strasse 1a, 1060 Vienna, Austria; philipp.doppler@tuwien.ac.at; 2 Research Division Phytochemistry and Plant Biochemistry, Institute of Chemical, Environmental, and Bioscience Engineering, TU Wien, Getreidemarkt 9, 1060 Vienna, Austria; christoph.kornpointner@tuwien.ac.at (C.K.); heidrun.halbwirth@tuwien.ac.at (H.H.); 3 School of Engineering, University of Applied Sciences Upper Austria, Stelzhamerstr. 23, 4600 Wels, Austria

**Keywords:** stirred photobioreactor, pigment composition, astaxanthin, adonixanthin, nitrogen starvation, salt stress, palmelloids, antioxidant capacity, fatty acid profile

## Abstract

We isolated a novel strain of the microalga *Tetraedron minimum* in Iceland from a terrestrial habitat. During long-term cultivation, a dish culture turned orange, indicating the presence of secondary pigments. Thus, we characterized *T. minimum* for growth and possible carotenoid production in different inorganic media. In a lab-scale photobioreactor, we confirmed that nitrogen starvation in combination with salt stress triggered a secondary carotenoid accumulation. The development of the pigment composition and the antioxidant capacity of the extracts was analyzed throughout the cultivations. The final secondary carotenoid composition was, on average, 61.1% astaxanthin and 38.9% adonixanthin. Moreover, the cells accumulated approx. 83.1% unsaturated fatty acids. This work presents the first report of the formation of secondary carotenoids within the family Hydrodictyaceae (Sphaeropleales, Chlorophyta).

## 1. Introduction

Microalgal cultures use greenhouse gas carbon dioxide (CO_2_) as the sole carbon source and the power of light for autotrophic growth. The generated biomass is a sustainable, literally green alternative for the production of various value-added compounds for biofuels [1], for health and nutrition [2], for pharmaceuticals [3], and other bioactive molecules, such as antibiotics [4].

A specific class of these compounds are carotenoids. Their structure derives from the tetraterpene lycopene, and approx. 850 different derivatives are reported today [5]. Carotenoids, which are known for their high antioxidant capacity, are naturally produced as primary compounds indispensable for photosynthesis or as secondary storage metabolites. For example, astaxanthin produced by *Haematococcus pluvialis* (Chlorophyta) finds extensive use in nutraceutical and pharmaceutical applications [6]. Distinct algae are reported to accumulate other carotenoids of special scientific and industrial interest, like adonixanthin, β-carotene, canthaxanthin, echinenone, lutein, or zeaxanthin [7,8]. However, to find natural producers of these carotenoids, more effort in the discovering and screening of new strains and species is needed [9,10,11]. In nature, the microbial accumulation of secondary carotenoids is done to secure cells during harmful environmental conditions, like nutrient starvation, excessive UV, or visible light radiation; extreme temperatures or desiccation; and specifically, during the formation of resting cell stages, like cysts [5,12]. Generally, it is not always evident if one specific abiotic factor or a combination of several ones is causing secondary carotenoid production. Therefore, the simulation of stress factors in the lab to force cells into carotenoid formation is tricky and sometimes impossible [13]. The most used and promising techniques are temperature changes, elevated light radiation, nitrate starvation, and osmotic stress by the addition of salt [14].

The alga evaluated in this work was collected in Iceland from a terrestrial location grown on wet, sun-exposed gravel and was identified as *Tetraedron minimum*. The species was first described by Hansgirg (1889), and the genus is named after its characteristic tetrahedral-shaped autospores [15,16].

The goal of this work was to characterize the novel strain of *Tetraedron minimum* to identify the stress factors responsible for its secondary carotenoid accumulation. Additionally, a profile of its fatty acids should be obtained. This study presents the first report of secondary carotenoid production within the green algal family of Hydrodictyaceae.

## 2. Materials and Methods

### 2.1. Sampling, Strain Isolation, and Identification

Macroscopic mats composed of several microalgae and cyanobacteria were sampled from gavel wet by dripping water close to a steep, sun-exposed slope (N63°42.144, W19°40.194, 157 m a.s.l.) at Þórsmörk, Iceland (11 July 2017). For the generation of unialgal strains, the community was inoculated on sterile agar plates (1.7% *w/v* Agar, plant cell culture tested, Sigma Aldrich, St. Lewis, MO, USA) either with 3N Bolt’s Basal Medium (3N-BBM) at pH 6.5 [17] or in Synthetic Freshwater Medium (SFM) at pH 6.2 [18]. The algae were kept at 15 °C and approx. 20 to 30 µmol photons m^−2^·s^−1^ and 14 h light/10 h darkness. Unialgal strains were obtained by picking colonies and transferring them to a new plate with a sterile loop and subsequent light microscopical control. The identification of strain WP156.1 tested in this study was confirmed by molecular means (18S rDNA marker), and the closest blast hit entry at the National Center for Biotechnology Information (NCBI) database was *Tetraedron minimum* var. *scrobiculatum* AY663042 (UTEX LB 1367; data not shown).

### 2.2. Light Microscopy

Light microscopical analysis of *T. minimum* and its different cell stages were performed with a Zeiss Axio Imager M1m (Carl Zeiss, Oberkochen, Germany) equipped with a Zeiss ×50/0.7 objective. The software AxioVision was used for imaging by an AxioCam MRc5 camera.

### 2.3. Cultivations

#### 2.3.1. Media

Bolt’s Basal Medium (BBM) contained 250 mg·L^−1^ NaNO_3_, 75 mg·L^−1^ MgSO_4_·7H_2_O, 25 mg·L^−1^ NaCl, 75 mg·L^−1^ K_2_HPO_4_, 175 mg·L^−1^ KH_2_PO_4_, 25 mg·L^−1^ CaCl_2_·2H_2_O, and 1 mL·L^−1^ trace metal mix [17]. The nitrogen content of 0.3N-BBM was the factor 0.3, with a final concentration of 75 mg·L^−1^ NaNO_3_. Likewise, the NaNO_3_ content of 3N-BBM was 750 mg·L^−1^. Other components were prepared like the original BBM. Synthetic Freshwater Medium (SFM) was made of 50 mg·L^−1^ Ca(NO_3_)_2_·4H_2_O, 50 mg·L^−1^ MgSO_4_·7H_2_O, 2.3 mg·L^−1^ K_2_HPO_4_, 30 mg·L^−1^ NaNO_3_, 19.2 mg·L^−1^ Na_2_CO_3_, and 1 mL·L^−1^ 1000× trace metal mix [18]. The 1000× trace metal mix stock was made of 8.82 g·L^−1^ ZnSO_4_·7H_2_O, 1.44 g·L^−1^ MnCl_2_·4H_2_O, 0.71 g·L^−1^ MoO_3_, 1.57 g·L^−1^ CuSO_4_·5H_2_O, 0.49 g·L^−1^ Co(NO_3_)_2_·6H_2_O, 11.4 g·L^−1^ H_3_BO_3_, 50.0 g·L^−1^ Na_2_EDTA·2H_2_O, and 4.98 g·L^−1^ FeSO_4_·7H_2_O [17]. Additionally, the media was supplemented with 1 mM HEPES and adjusted to pH 6.5 by 0.1 M NaOH prior to autoclaving. Subsequently, vitamins were added aseptically via syringe and a 0.22 μm filter to a final concentration of 1.0 mg·L^−1^ vitamin B1, 0.25 mg·L^−1^ vitamin H, and 0.15 mg·L^−1^ vitamin B12 [17].

#### 2.3.2. Cultivations in Erlenmeyer Flasks

Shake flask cultures for initial growth experiments were conducted in 250 mL Erlenmeyer flasks. Each flask was filled with 50 mL of either BBM, 0.3N-BBM, or SFM. More flasks containing the three different media were prepared and supplemented with 10 or 30 mM NaCl. The experiment was started by the addition of 1 mL preculture grown in SFM with an optical density (OD_600_) of approx. 0.50. Then, they were placed in a Minitron incubation shaker (Infors, Basel, Switzerland) at 20 °C with a set-point of the photosynthetic photon flux density (PPFD) of 20 μmol·m^−2^·s^−1^ during a 14 h light/10 h darkness cycle with occasional manual shaking. The experiment was run for 30 days for growth characterization.

#### 2.3.3. Cultivations in Stirred Photobioreactor

A Ralf multi-bioreactor system (Bioengineering, Wald, Switzerland) with 2.0 L total volume per reactor was equipped with two 55 mm turbine impellers for stirring. The glass vessel had a glass jacket for heating and cooling. Illumination was provided externally via a fluorescent light bulb. The inner diameter of the reactor was 95 mm, and the illumination in the center was 100 μmol·m^−2^·s^−1^, measured in a liquid medium by an ULM-500 with an US-SQS/L sensor (Walz, Ulm, Germany). An EasyFerm Plus pH probe (Hamilton, Bonaduz, Switzerland) was installed. Aeration of air and pure CO_2_ were provided by two separate type 4850 mass flow controllers operated via a 0254 controller (Brooks Instruments, Hatfield, PA, USA).

SFM medium (1.25 L) was poured into the vessel prior to autoclaving. After that, a vitamin solution was applied aseptically by syringe through a sterile 0.22 μm filter via a septum. The medium was aerated by 3.0% CO_2_-enriched air, which resulted in pH 6.4 ± 0.1 in the medium. The stirrer was set to 300 rpm and the temperature controlled at 20 °C. A preculture grown in SFM was used for inoculation to a final OD_600_ in the reactor of approx. 0.03 via septum. To eliminate different preculture conditions, the experiment and sampling was started when OD_600_ reached 0.10 ± 0.01. Sampling was carried out 3 times a week every 2 to 3 days with additional weekly 100 mL samples for the biomass harvest to determine the pigment composition and antioxidant capacity. OD_600_ measurements for monitoring cell growth were carried out in 1 mL cuvettes on a Nanodrop One photometer (Thermo Fisher Scientific, Waltham, MA, USA). The dry cell weight (DCW) of the lyophilized biomass was determined gravimetrically at the end of fermentation, and the following correlation to the OD_600_ could be found:

DCW [mg·L^−1^] = 272.5 · OD_600_ (1)

### 2.4. Determination of Nitrate Concentration

After centrifugation, the supernatant of 1 mL culture was directly used for NO_3_^−^ concentration measurements on an ICS-6000 ion chromatography (IC) system (Thermo Fisher Scientific, Waltham, MA, USA). A Dionex IonPac AS11 with guard column (Thermo Fisher Scientific, Waltham, MA, USA) was set to 30 °C and operated with a flow of 2.0 mL·min^−1^. Mobile phase A was 100% H_2_O and phase B 100 mmol·L^−1^ NaOH. The binary gradient was from 0 to 1 min 0.2% B, from 1 to 6 min to 5% B, from 6 to 13 min increase to 24% B, from 13 to 13.5 min set to 38% B, and hold until 14.5 min. After this, the initial 0.2% B was reached after 15 min and held until 20 min for equilibration before the next injection. A conductivity detector combined with AERS suppressor (Thermo Fisher Scientific, Waltham, MA, USA) was used for analyte detection. Nitrate standards (Sigma Aldrich, St. Lewis, MO, USA) were prepared and used for quantification of the samples. Data was logged and evaluated by Chromeleon 7.2.8 (Thermo Fisher Scientific, Waltham, MA, USA).

### 2.5. Determination of Carbohydrate Content

Carbohydrate quantification was done similar as described by [19]. The cell pellet of 1 mL culture was dried at 75 °C for 24 h. 1 mL of 7.5% (*v/v*) H_2_SO_4_ in H_2_O was added, and the sample was placed on a heating block at 95 °C, 900 rpm for 120 min. Insoluble particles were separated by centrifugation at 4 °C and 21,000× *g* for 10 min. The sample (100 μL) was diluted by the addition of 400 μL H_2_O. Glucose (Sigma Aldrich, St. Lewis, MO, USA) was treated accordingly and used for calibration standards. The measurements were done on an ICS-6000 IC system (Thermo Fisher Scientific, Waltham, MA, USA). The stationary phase was a Dionex CarboPac PA10 combined with amino trap column (Thermo Fisher Scientific, Waltham, MA, USA) heated to 30 °C. The mobile phase (1.0 mL·min^−1^) was pumped as 18 mmol·L^−1^ NaOH for 15 min, followed by a 200 mmol·L^−1^ NaOH for 10 min and an 18 mmol·L^−1^ NaOH equilibration step for another 35 min before the next sample was injected. An electrochemical (EC) detector was operated. Data was evaluated and quantified by the software Chromeleon 7.2.8 (Thermo Fisher Scientific, Waltham, MA, USA).

### 2.6. Pigment Extraction, Identification, and Quantification

#### 2.6.1. Pigment Extraction

About 20 mg lyophilized biomass was filled into screw-capped reaction tubes. One 5 mm and 0.3 g of 1 mm glass beads were added. The tubes were cooled down in liquid nitrogen (LN2) for 10 min before disruption using a FastPrep-24 Instrument (MP Biomedicals, Santa Ana, CA, USA). The settings for this device were 6.0 m·s^−1^ for 45 s. In total, three runs were done, whereas 1 mL acetone was added prior to the third homogenization run. In between the sample, tubes were cooled down with LN2. Then, the mixture was mixed, and cell debris and acetone extract were separated by centrifugation for 10 min at 10,000× *g*. The acetone phase was transferred into 5 mL volumetric flasks by pipette. This procedure was repeated three times. The work was done under dimmed light. Samples were filtered through 0.22 μm filters before high-performance liquid chromatography (HPLC) analysis.

#### 2.6.2. Pigment Identification

The pigments were identified by high-performance liquid chromatography coupled to mass spectrometry (HPLC-MS). Acetone extracts were injected on a 1290 Infinity II LC System (Agilent Technologies, Santa Clara, CA, USA) equipped with diode array detector (DAD) at 450 nm. An Acclaim C30, 3 μm, 2.1 × 100 mm² column (Thermo Fisher Scientific, Waltham, MA, USA) was used for separation at 20 °C. The mobile phases were premixed as buffer A: 91% methanol (MeOH), 5% methyl *tert*-butyl ether (MTBE), 3.9% H_2_O, and 0.1% formic acid and buffer B: 46% MeOH, 50% MTBE, 3.9% H_2_O, and 0.1% formic acid. The total flow was 0.3 mL·min^−1^, and the following gradient was operated: from 0 to 6 min 1% B, from 6 to 24 min 100% B, which was held for 4.5 min. From 28.5 to 29 min, back to 1% B, which was held for 7 min for equilibration prior the next injection. Total run time was 36 min. Mass detection was done by a 6545 LC/Q-TOF mass spectrometer (Agilent Technologies, Santa Clara, CA, USA) equipped with a multimode ion source operated in atmospheric pressure chemical ionization (APCI), positive mode. Masses from 100 to 1700 *m/z* were recorded.

Identification was done by the software Mass Hunter Qualitative Analysis 10.0 (Agilent Technologies, Santa Clara, CA, USA). Characteristic spectral absorption of each peak and the theoretical and detected ion masses at the same retention time were compared [20]. Astaxanthin and adonixanthin monoester (ME) and astaxanthin diester (DE) were identified via characteristic absorption spectra in comparison to their unesterified forms [21].

#### 2.6.3. Pigment Quantification

Quantification of carotenoids was carried out on a Vanquish Flex HPLC system (Thermo Fisher Scientific, Waltham, MA, USA). The same column, flow rate, eluents, and gradients as for HPLC-MS measurements were used. However, detection was done by DAD at 450 nm. Pure astaxanthin (Sigma Aldrich, St. Lewis, MO, USA) was dissolved in acetone and used as external standard. All carotenoids were quantified in their absorption in reference to astaxanthin at 450 nm and expressed in μmol·g^−1^ lyophilized biomass. Device operation and data handling was done with Chromeleon 7.2.8 (Thermo Fisher Scientific, Waltham, MA, USA).

Chlorophyll *a* and *b* were quantified as described by [22]. One milliliter of acetone extract was diluted with 0.25 mL of 2.5 mM NaH_2_PO_4_ buffer at pH = 7.8 to prepare samples in aqueous 80% acetone. Subsequently, absorption of these extracts at 647, 664, and 750 nm was recorded on a Nanodrop One photometer (Thermo Fisher Scientific, Waltham, MA, USA). Chlorophyll *a* and *b* concentrations were calculated by Formulas 7 and 8 in [22].

### 2.7. Antioxidant Capacity Assay

The antioxidant activity of the extracts was analyzed using the Ferric-reducing antioxidant power (FRAP) assay. In this assay, Fe^3+^ is reduced to Fe^2+^ by the antioxidant compounds and forms a colored complex with 2,4,6-tripyridyl-*s*-triazine in acetate buffer (pH 3.6) at 593 nm [23]. For the assay, the reactive solution was freshly prepared with 25 mL of 300 mM acetate buffer, pH 3.6, and 2.5 mL of 20 mM FeCl_3_·6H_2_O in deionized water, as well as 2.5 mL of 10 mM 2,4,6-tripyridyl-*s*-triazine in 40 mM HCl. The FRAP working solution (300 µL) was mixed with the acetonic algae extract (30 µL), and after 30 min, the absorbance was recorded by a SPECTROstar Nano absorbance microplate reader (BMG LABTECH, Ortenberg, Germany). The calibration was performed with astaxanthin (Sigma Aldrich, St. Lewis, MO, USA) in acetone, and the results are expressed in astaxanthin equivalent μmol_Ax_·g^−1^ lyophilized biomass.

### 2.8. Lipid Extraction, Preperation, and Analysis of Fatty Acids

Lipid extraction by chloroform and methanol was done as described by [24]. Approx. 50 mg lyophilized biomass was homogenized as described for pigment extractions. Instead of acetone for the third round of homogenization, 1 mL of chloroform:methanol = 2:1 (*v/v*) was added. Derivatization due to HCl catalyzed transesterification in methanol to prepare fatty acid methyl esters (FAMEs) was done, as shown by [25]. The reaction was done at 45 °C for 16 h in an incubation shaker at 180 rpm. The organic phase was directly used for gas chromatography coupled to a mass spectrometer (GC-MS) analysis.

The 7890A/5975C GC-MS system (Agilent Technologies, Santa Clara, CA, USA) was equipped with a CTC Combi PAL autosampler. The gradient was slightly adopted from [26]. Helium carrier gas (1.04 mL·min^−1^) was pushed through a Permabond FFAP capillary column with 30 m length, 0.25 mm inner diameter, and 0.25 μm film thickness (Macherey-Nagel, Düren, Germany). The oven-heating program was initially 50 °C for 2 min, followed by a 10 °C·min^−1^ increase to 200 °C, which was held for 10 min. Then, another 10 °C·min^−1^ ramp to 220 °C was done and held constant for 15 min. The transfer line temperature was 280 °C, and the flow was split 1:5 to measure simultaneously the quantity of FAMEs via a flame ionization detector (FID) and the corresponding mass values via a MS detector. For the mass detector, the source temperature was 230 °C, electron energy 70.3 eV, and the scanning range was from 35 to 750 *m/z*. The identification of each FAME was confirmed by a reference library search with Mass Hunter Qualitative Analysis (Agilent Technologies, Santa Clara, CA, USA).

## 3. Results

### 3.1. Preleminary Experiments for Adequate Cultivation Conditions

After isolation, *Tetraedron minimum* was transferred to SFM and 3N-BBM plates. During long-time exposure for eight months in a light incubator at 15 °C, the culture at 3N-BBM turned orange, indicating the accumulation of secondary pigments. In contrast, the SFM plate stayed green (Supplementary Materials, Figure S1). These two plates differed in terms of chemical media composition, with an 11.6-fold higher NO_3_^−^ content of 3N-BBM. Due to a higher total salt content in 3N-BBM, the electrical conductivity raised almost 3.3-fold to 1627 μS·cm^−1^ versus 499 μS·cm^−1^ for SFM.

**Figure 1:**
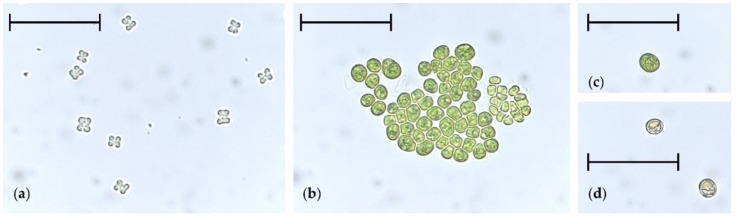
Different cell types of *Tetraedron minimum* found in liquid cultures. (**a**) Tetrahedral autospores, (**b**) cell aggregates, (**c**) green cysts (image taken after 3 weeks of cultivation), and (**d**) orange cysts showing reddish secondary pigmentation (image taken at the end of cultivation). Scale bar is 50 μm.

Growth experiments in Erlenmeyer flasks were conducted to see how the electrical conductivity, reflecting the amount of total dissolved solids, influences the growth of *T. minimum* and if high salt concentrations could be an appropriate stress factor for a subsequent photobioreactor (PBR) cultivation of *T. minimum* (Supplementary Materials, Table S1). After 30 days, variations in the growth and morphology of *T. minimum* cells were observed (Supplementary Materials, Figure S2). In SFM, the cells were evenly spread, indicated by the homogeneous green coloration of the whole culture. Using 0.3N-BBM led to cell agglomeration. In BBM, the biomass content and the size of agglomerates in the flask appeared less compared to 0.3N-BBM. Light microscopical observations of SFM cultures without additional NaCl showed cell packages in the form of autospores (Figure 1a). Flasks containing higher amounts of dissolved salts resulted in agglomerates consisting of roundish cells with aggregate diameters of more than 50 μm (Figure 1b). These experiments showed that *T. minimum* was sensitive to the total salt content of the culture medium.

### 3.2. Photobioreactor Cultivations

**Figure 2:**
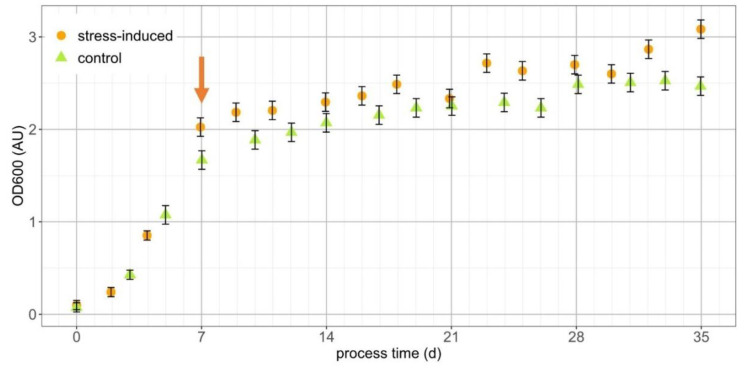
Optical density (OD_600_) of *T. minimum* cultivations. The orange arrow indicates the time of NO_3_^−^ depletion and the point of NaCl addition (for stress-induced secondary carotenoid accumulation). NaCl supplementation was omitted in the control run.

For the investigation of accumulated secondary carotenoids in *T. minimum*, a combination of nitrogen starvation, as well as salt stress, in 1.25 L stirred PBR cultivations was induced, and the growth was monitored (Figure 2). Therefore, a two-step cultivation process with an initial biomass generation phase in SFM was done until NO_3_^−^ was depleted after seven days. Consequently, a salt stress phase was started by a bonus addition of NaCl to a final concentration of 30 mM. For control purposes, a PBR containing the same starting media without added NaCl was operated.

During four weeks of nitrogen starvation, the OD_600_ of the stress-induced culture raised from 2.03 to 3.08 (+52%) compared to an increase from 1.67 to 2.47 (+48%) in the control. In addition, *T. minimum* cells turned into green (Figure 1c) and, solely in the stressed culture, orange cysts with definite, orange-colored cytosolic compartments, indicating accumulated secondary carotenoids (Figure 1d). The diameters of the more-or-less sphere-shaped cysts were between 10 and 15 μm. The culture in the control PBR had a yellow-greenish coloration after the same process time.

The final dry cell weight (DCW) after harvest was 760 ± 18 mg·L^−1^ for the stressed and 708 ± 20 mg·L^−1^ for the control culture. In relation to the measured starting NO_3_^−^ levels of 46.1 ± 0.4 mg·L^−1^ and 45.9 ± 0.4 mg·L^−1^, the biomass production yield per spent NO_3_^−^ was 16.5 ± 0.5 mg·mg^−1^ and 15.4 ± 0.6 mg·mg^−1^, respectively. The carbohydrate content per cell was 16.2% ± 0.5% and 12.9% ± 0.5% of DCW.

### 3.3. Pigment Composition

**Figure 3:**
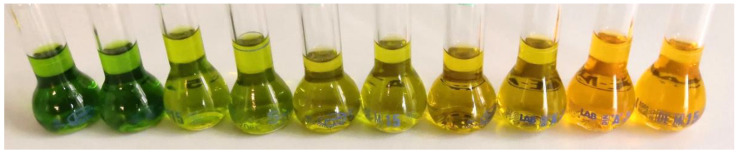
Extracts in acetone prepared from weekly samples during stress-induced photobioreactor (PBR) cultivations for the quantitative evaluation of pigments and the antioxidant capacity. From left, samples in duplicates from the end of week 1 to 5.

**Figure 4:**
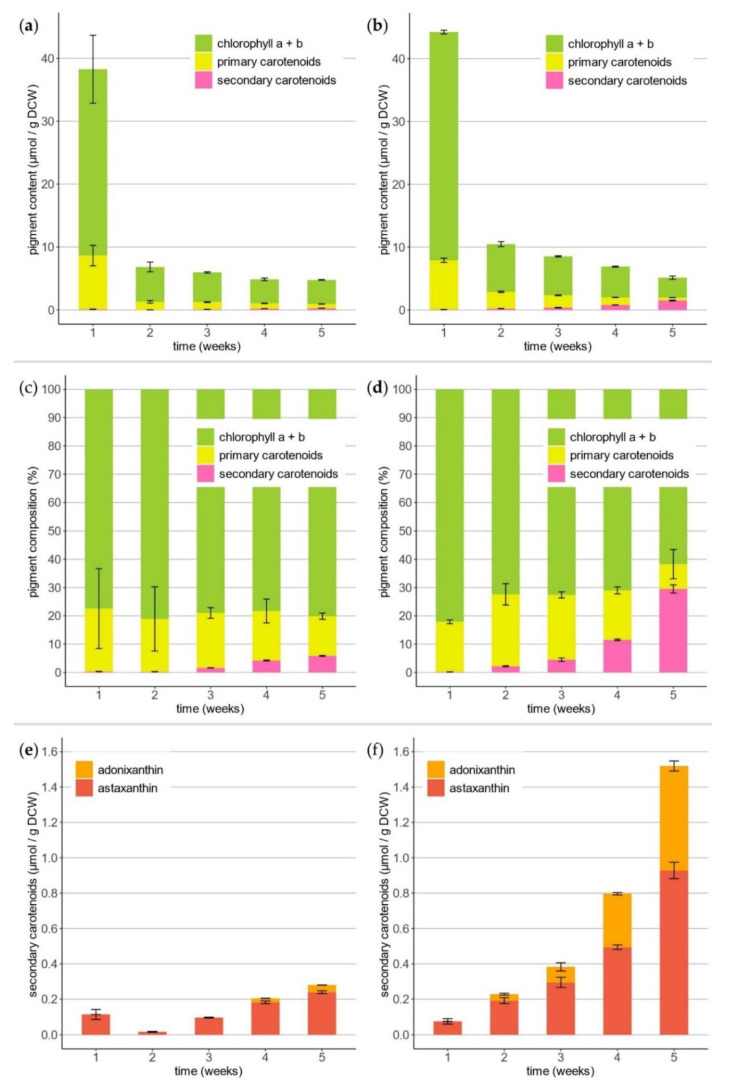
Quantitative evolution of pigments over the course of 5-week cultivation processes of *T. minimum*. (**a**,**b**) Total content of pigments, (**c**,**d**) molar composition of pigments, and (**e**,**f**) accumulated secondary carotenoids astaxanthin and adonixanthin, including the esterified forms. (**a**,**c**,**e**) Control and (**b**,**d**,**f**) salt stress-induced PBR.

The orange-colored biomass harvested at the end of the *T. minimum* PBR cultivations was used for qualitative analysis of the pigments by HPLC-MS (Supplementary Materials, Figure S3 and Table S2). We identified chlorophyll *a* and *b*; the primary carotenoids neoxanthin, violaxanthin, lutein, zeaxanthin, canthaxanthin, echinenone, and β-carotene; and the secondary carotenoids astaxanthin and adonixanthin. In addition, weekly samples were drawn for pigment extraction (Figure 3) and subsequent quantification by HPLC (Figure 4).

After the first week, chlorophyll *a* and *b* comprised 36.31 ± 0.29 μmol·g^−1^ biomass in the designated salt-stressed and 29.62 ± 5.41 μmol·g^−1^ in the control reactor (Figure 4a,b). During the following four weeks of nitrogen starvation, the total chlorophyll content declined significantly to 3.18 ± 0.27 μmol·g^−1^ and 3.83 ± 0.05 μmol·g^−1^ at the end of cultivation. The sum of the primary carotenoids also decreased from 7.84 ± 0.33 μmol·g^−1^ to 0.45 ± 0.01 μmol·g^−1^ and 8.53 ± 1.62 μmol·g^−1^ to 0.67 ± 0.03 μmol·g^−1^, respectively. In contrast, the amount of secondary carotenoids increased from 0.07 ± 0.02 μmol·g^−1^ to 1.52 ± 0.07 μmol·g^−1^ compared to the control with 0.11 ± 0.03 μmol·g^−1^ to 0.28 ± 0.01 μmol·g^−1^ (Figure 4e,f). A relative content of 61.1 ± 8.6% astaxanthin and 38.9 ± 5.4% adonixanthin (including esterified derivates) regarding secondary carotenoids was determined in the salt stress-induced process. The molar pigment composition showed relatively low amounts of primary and secondary carotenoids in relation to chlorophyll *a* and *b* in all samples (Figure 4c,d).

### 3.4. Antioxidative Capacity

The antioxidant capacity was measured using the Ferric ion-reducing antioxidant power (FRAP) assay (Table 1). The highest values were observed at the beginning in both PBRs with 58.4 ± 4.8 μmol_Ax_·g^−1^ and 36.2 ± 2.5 μmol_Ax_·g^−1^, respectively. After the biomass growth phase ended, the antioxidant capacity dropped rapidly. No clear difference in the end samples of stress-induced and control cultures with 18.5 ± 1.1 μmol_Ax_·g^−1^ and 18.3 ± 1.7 μmol_Ax_·g^−1^ was observed.

### 3.5. Fatty Acid Profile

For the total lipid extraction, a green-colored *T. minimum* biomass of the preliminary cultivations without induced stress was used as a representative biomass sample. The fatty acid (FA) profile of this *T. minimum* sample is given in Table 2. FAs with 14 to 22 carbon atoms were observed. The most abundant saturated form was C16:0 (palmitic acid) with 13.7% ± 2.8%. Monounsaturated C18:1 (oleic acid) was the most abundant form with 56.7% ± 6.1% of all FAs. The most abundant polyunsaturated FA was C18:3 (α-linolenic acid) with 8.4% ± 1.6%. The quadruple unsaturated FA C18:4 (stearidonic acid) with 2.3% ± 1.0% was the highest concentrated unsaturated form. In total, 60.1% ± 7.6% monounsaturated and 23.0% ± 6.9% polyunsaturated FAs were detected. 

## 4. Discussion and Conclusions

### 4.1. Preliminary Experiments

During long-term Petri dish cultivation, the cells of *T. minimum* (strain WP156.1) turned orange, indicating the production of secondary carotenoids. This was surprising, since no report of this ability for any member of Hydrodictyaceae has been published before. However, Fučiková et al. (2014) described several other members from different families of the order Sphaeropleales, where strains from similar terrestrial habitats had old cells that turned reddish, as well [27]. Remarkably, only the culture growing on Bolt’s Basal Medium with a triple nitrate content turned orange during our study, whereas the Synthetic Freshwater Medium plate stayed green. After the evaluation and analysis of the media ingredients, two major differences were identified [17,18]. Firstly, 3N-BBM contained almost 12 times the amount of NO_3_^−^, and secondly, the measured electrical conductivity (EC) was 1627 μS·cm^−1^ and only 499 μS·cm^−1^ for SFM. Converted and expressed in total dissolved solids, this meant 13.2 mM and 2.8 mM NaCl equivalents [28,29]. Excessive NO_3_^−^ availability is not regarded as a stress factor but is even thought to prevent secondary carotenoid accumulation [13]. However, this compound, in combination with other composition differences of the media, caused a higher osmotic pressure of approx. 10 mM NaCl equivalents. This correlates with the reported values for the salt stress-induced secondary carotenoid production of green algae in the literature [30,31,32]. Consequently, these findings indicated that nitrogen starvation during the long-term Petri dish cultivations could not be the only stress factor, and salt stress most likely was—in combination with nitrogen starvation—the responsible trigger for secondary carotenoid production.

Consequently, experiments in Erlenmeyer flasks with different media and salt contents were conducted. After 30 days of incubation, clear differences were observed by visual inspection. The lowest salt-containing medium SFM showed the highest biomass content homogeneously distributed in the flask. Microscopic observations of the biomass grown in 0.3N-BBM and BBM showed roundish, agglomerated cells instead of characteristic tetrahedral-shaped autospores [15,16]. This morphological phenomenon of “palmelloid” stage formation was previously described for *Chlamydomonas* spp. and was directly ascribed to salt-induced stress [33,34]. In addition, this indicated a salt stress-induced secondary carotenoid formation in *T. minimum*.

### 4.2. Photobioreactor Cultivations

A classical two-phase strategy was performed for culturing *T. minimum* [35,36,37]. In the first stage, the optimum conditions for growth with low osmotic pressure were provided by SFM. The growth phase started with the initial growth rates (μ_max_) of 0.53 ± 0.06 d^−1^ in both reactors. After seven days, minor differences in the OD_600_ were observed. In both cases, nitrate was completely consumed, and salt stress was applied to the designated stress-induced culture. This initiated a four-week stress phase with the average weekly growth rates, μ, of 0.105 w^−1^ in the stressed culture and 0.098 w^−1^ for the control. Only the culture with combined nitrogen starvation and salt stress turned orange, at the end.

High levels of chlorophyll *a* and *b* and the primary carotenoids were quantified at the end of the growth phase. The pigment concentrations dropped rapidly when nitrogen stress set in. After one week, the absolute pigment loss in the salt stress culture was 76.3% ± 2.2% and 81.0% ± 8.6% in the control. Therefore, this effect was independent of salt stress. The decrease of the pigment content was directly ascribed to nitrogen starvation stress [38,39]. Due to a nitrogen deficiency, the cells started to develop a cyst stage. This process is associated with the loss of large amounts of the total chlorophyll and, also, primary carotenoids [12]. An ongoing decrease in chlorophyll *a* and *b* and the primary carotenoids was observed until the end of the cultivation but to a lower extent. The same rapid drop of the chlorophyll and primary carotenoids after nitrogen starvation was already shown in comparable nitrogen-limited *H. pluvialis* cultivations [39]. Vice versa, secondary carotenoids were produced and accumulated. From week two to five, the total content almost doubled every week, to a final amount of 1.52 ± 0.07 μmol·g^−1^. In the control, the concentration only reached 0.28 ± 0.01 μmol·g^−1^. Moreover, the control culture did not turn orange after four more weeks of cultivation, clearly indicating that a combination of nitrogen starvation and salt stress was responsible for the secondary carotenoid accumulation.

The antioxidant capacity of the biomass extracts showed the highest values at the end of the growth phase and dropped significantly after one week of nitrogen starvation. Photosynthesis requires antioxidants in the form of primary carotenoids for chlorophyll quenching and to avoid radical oxygen species (ROS) [40,41]. This trend was caused by the weekly decline of the total chlorophyll and primary carotenoid contents, which were explained by cyst formation due to nitrogen starvation. The synthesis of secondary carotenoids in the late phases did not lead to a significant impact on the reduction power of the extracts. This was ascribed to the low absolute quantity of secondary carotenoids of only 0.091 ± 0.004 wt% of the biomass content. Additionally, the antioxidant capacity is influenced by many other molecules likely present in considerable amounts in the microalgae biomass, such as α-tocopherol, phenolic compounds or unsaturated FAs [42,43,44]. The FA profile revealed a remarkably high content of mono- and polyunsaturated FAs with a total ratio of 83.1% ± 14.5%.

### 4.3. Secondary Carotenoids

The main secondary carotenoids of *T. minimum* present at the end of fermentation were astaxanthin and adonixanthin in free and esterified forms. Similar compositions were reported for other green algae (cf. *Chlorococcum*) [45,46], but generally, sources rich in adonixanthin are rarer than the quite common astaxanthin. The third-largest peak in the chromatogram was a coelution of lutein and zeaxanthin; no quantitative discrimination could be done due to same theoretical molecule mass and overlapping absorption spectra of the two substances [47]. However, zeaxanthin is an intermediate in the biosynthetic pathways of secondary carotenoids accumulated by *T. minimum* from β-carotene via zeaxanthin and adonixanthin to astaxanthin, with a subsequent esterification with FAs [5,39].

In conclusion, this is the first study describing secondary carotenoid production in *T. minimum* and, also, the first positive report regarding the Hydrodictyaceae. Particularly, the abundant occurrence of adonixanthin was remarkable for a green microalga [48]. Finally, insights were given about the stress mechanisms and the kinetics of secondary carotenoid formation, and future methodological optimizations have to aim for an increase in the yield.

## Figures and Tables

**Table 1:** Antioxidant capacities of *T. minimum* extracts, given as astaxanthin equivalents (μmol_Ax_).

Process Time (Weeks)	Control (μmol_Ax_·g^−1^)	Stress-Induced (μmol_Ax_·g^−^^1^)
1	36.2 ± 2.5	58.4 ± 4.8
2	22.2 ± 0.1	24.0 ± 1.4
3	18.0 ± 0.7	22.8 ± 0.1
4	17.5 ± 0.7	19.8 ± 0.1
5	18.3 ± 1.7	18.5 ± 1.1

**Table 2:** Relative fatty acid contents of the *T. minimum* biomass at the end of the stress-induced cultivation.

Fatty Acid	Relative Content (%)
C14:0	0.1 ± 0.1
C16:0	13.7 ± 2.8
C16:1 (7Z or 9Z) *	1.2 ± 1.2
C16:2 (7Z, 10Z)	0.9 ± 0.7
C16:3 (7Z, 10Z, 13Z)	5.0 ± 1.7
C16:4 (4Z, 7Z, 10Z, 13Z)	0.6 ± 0.4
C18:0	2.6 ± 0.7
C18:1 (9Z or 11Z or 13Z) *	56.7 ± 6.1
C18:2 (9Z, 12Z)	5.9 ± 1.6
C18:3 (9Z, 12Z, 15Z)	8.4 ± 1.6
C18:4 (6Z, 9Z, 12Z, 15Z)	2.3 ± 1.0
C20:0	0.2 ± 0.2
C20:1 (9Z or 11Z or 13Z) *	2.2 ± 0.3
C22:0	0.3 ± 0.2
Total saturated	16.9 ± 4.1
Total monounsaturated	60.1 ± 7.6
Total polyunsaturated	23.0 ± 6.9

* Isomers were not discriminated.

## Data Availability

The data presented in this study are available on request from the corresponding authors.

## Supplementary Materials

The following are available online at https://www.mdpi.com/2075-1729/11/2/107/s1: Figure S1: Plate cultures. Figure S2: Growth experiments in Erlenmeyer flasks. Figure S3: Chromatogram (450) nm of an acetonic extract for HPLC-MS. Table S1: Electrical conductivity of the media for growth experiments. Table S2: Identified pigments in the biomass extract.
