# Supplementary material for: Tetraedron minimum, First Reported Member of Hydrodictyaceae to Accumulate Secondary Carotenoids
Source: Life (Basel). 2021 Jan 30;11(2):107. doi: 10.3390/life11020107 (PMC7911234; doi:10.3390/life11020107)
Supplement: Supplementary file 1 [file life-11-00107-s001.pdf]

life

MDPI

Supplementary Information

Tetraedron minimum, First Reported Member of Hydrodictyaceae to Accumulate Secondary Carotenoids

Philipp Doppler, Christoph Kornpointner, Heidi Halbwirth, Daniel Remias and Oliver Spadiut

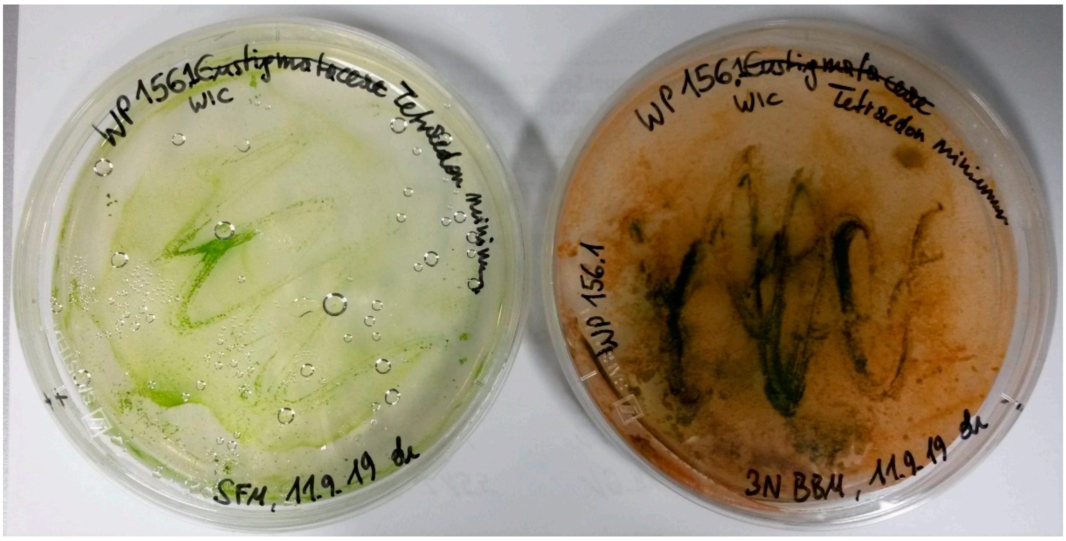

Figure S1. *T. minimum* growing on SFM (left, stayed green) and 3N-BBM plate (right, turned reddish). Picture was taken 8 months after inoculation.

Table S1. Electrical Conductivity of media prepared for pre-experiments with varying NaCl levels.

Medium	NaCl added (mM)	El. Conductivity (µS·cm^{-1})	NaCl Equiv. (mM)
	0	883	6.3
**BBM**	10	2043	17.1
	30	4669	41.8
	0	622	3.9
**0.3N-BBM**	10	1841	15.2
	30	4185	37.2
	0	499	2.8
**SFM**	10	1668	13.6
	30	3960	35.0

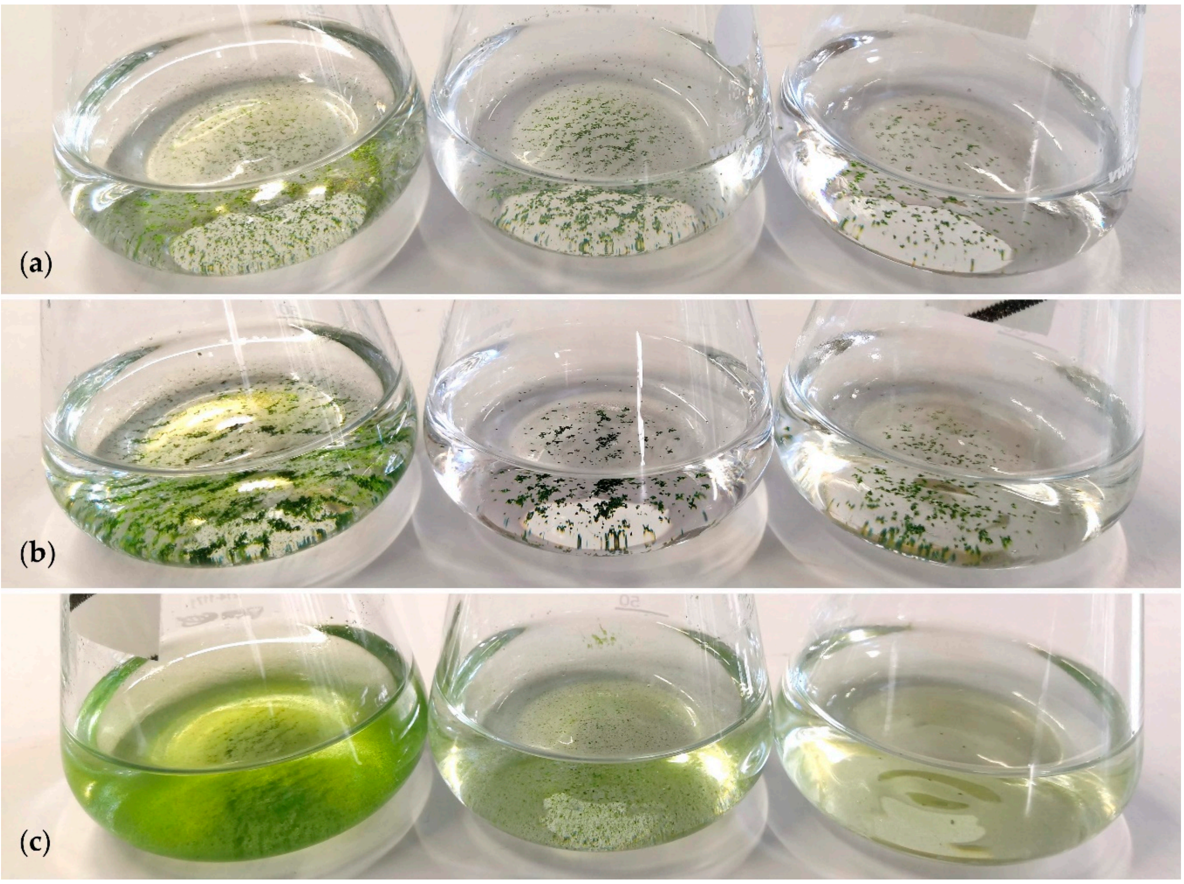

Figure S2. Growth experiments of *T. minimum* in Erlenmeyer flasks with three different media (**a**) BBM (**b**) 0.3N-BBM (**c**) SFM are shown with additional NaCl at concentrations of 0, 10 and 30 mM (from left to right). Pictures were taken 30 days after inoculation.

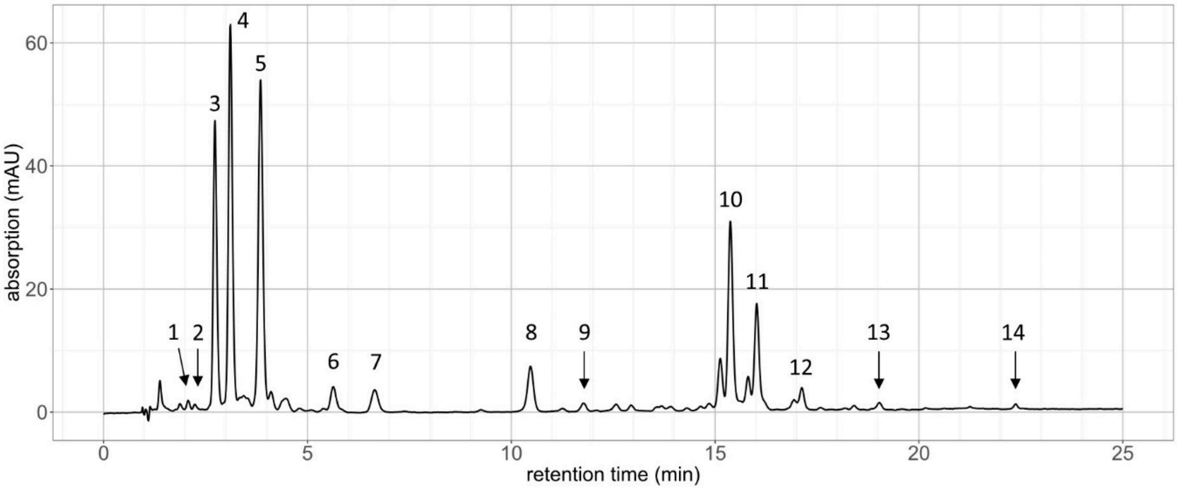

Figure S3. DAD-chromatogram at 450 nm of HPLC-MS analysis of an orange-colored *T. minimum* extract. Peak assignments are shown in Table S2.

Table S2. Identified pigments by HPLC-MS in the *T. minimum* extract.

	Retention (min)	Assigned Substance	Detected Mass $(M+H)^+$	Theoretical Mass $(M+H)^+$	Mass Error (ppm)
1	2.1	neoxanthin	601.4233	601.4251	3.0
2	2.2	violaxanthin	601.4229	601.4251	3.7
3	2.7	astaxanthin	597.3936	597.3938	0.3
4	3.1	adonixanthin	583.4124	583.4146	3.8
5	3.9	lutein + zeaxanthin *	569.4345	569.4353	1.4
6	5.7	canthaxanthin	565.4039	565.4040	0.2
7	6.7	chlorophyll *b*	907.5202	907.5219	1.9
8	10.5	chlorophyll *a*	893.5401	893.5426	2.8
9	11.8	echinenone	551.4238	551.4247	1.6
10	15.4	astaxanthin ME			
11	16.0	adonixanthin ME			
12	17.1	astaxanthin ME			
13	19.0	β-carotene	537.4416	537.4455	7.3
14	22.4	astaxanthin DE			

* co-eluting isomers, ME = monoester, DE = diester
